# eDNA-based detection of the invasive crayfish *Pacifastacus leniusculus* in streams with a LAMP assay using dependent replicates to gain higher sensitivity

**DOI:** 10.1038/s41598-022-10545-w

**Published:** 2022-04-21

**Authors:** David Porco, Sylvie Hermant, Chanistya Ayu Purnomo, Mario Horn, Guy Marson, Guy Colling

**Affiliations:** Life Science Department-Invertebrate Zoology, Population Biology and Evolution / Fondation Faune Flore, Musée National d’Histoire Naturelle, 24, Rue Münster, 2160 Luxembourg, Luxembourg

**Keywords:** PCR-based techniques, Invasive species

## Abstract

LAMP assays are becoming increasingly popular in the field of invasive species detection but are still underused in eDNA-based monitoring. Here, we propose a LAMP assay designed to detect the North American crayfish species *Pacifastacus leniusculus* in water samples from streams. The presence of *P. leniusculus* was detected through this new LAMP assay in all but one of the nine sites sampled. No correlation was found between ddPCR absolute concentration measurements and the number of LAMP-positive technical replicates. However, we showed that using dependent technical replicates could significantly enhance the detection sensitivity of the LAMP assay. Applied to other assays, it could improve sensitivity and thus allow for a more efficient use of eDNA-based LAMP assays for invasive species detection in aquatic ecosystems.

## Introduction

Developed by Notomi et al.^1^ Loop-mediated isothermal amplification (LAMP) allows for highly specific and sensitive target detection. It operates under isothermal conditions thanks to the strand displacement activity of specific DNA polymerases (Bst). The speed of the reaction was further improved by the introduction of Loop primers^2^. The technique was originally applied in the medical area for the detection of pathogens in the field or at the point of care^3–5^. Its simplicity and low operating cost make this technique an important asset when resources are limited. Hence, even if it is still increasingly used in the clinical detection of human pathogens (e.g., ^6–9^), assays have also been developed in various other fields, such as pathogen detection in crops^10–12^, in aquaculture^13^, in food safety^14,15^ or in food market control for species identification^16^.

One of the later applications of LAMP assays is the detection and identification of invasive species of invertebrates^17–21^. Moreover, it allowed to distinguish between different cryptic biotypes within an invasive pest species that exhibited different ecology, host range and pesticide resistance^22^. The LAMP method is particularly relevant for this application, as it is cost effective, rapid and can be operated by agents with very limited training^23^.

However, most of these assays were operated from extracts originating from larvae, adult specimens, eggs or direct products such as feces. This implies the search and handling of specimen samples that can be time consuming. Advances have been made in using LAMP assays to process environmental samples such as hive debris^24^ and water samples^23,25,26^. Three of the LAMP studies based on eDNA detection in water samples were targeted at mussel species from the *Dreissena* genus^23,26^, and one was developed to detect the presence of a parasite’s intermediate host (a snail species) in drinking spots for livestock^25^. The coupling of eDNA sampling with a LAMP detection approach was thus proven operational. However, (1) those assays targeted high-density population mussel species that shed high amounts of DNA into the environment through external fecundation and (2) the snail survey was undertaken in small water volumes with low stream velocity, thus limiting the impact of the dilution factor^25,26^.

Here, we propose to test eDNA-based detection with a LAMP assay targeted at a crayfish species; these are known to exhibit low DNA shedding^27,28^ Moreover, we tested this approach with actual field conditions that could increase the difficulty of detection i.e. high/medium stream velocity. We also wanted to test the use of field-practical volumes for water sample replicates (i.e. less than one liter) as, with those, populations exhibiting but a few individuals, thus shedding lower DNA amounts, could be overlooked^23^. In this case, one strategy is to increase the volume of the replicates filtered (e.g.^29,30^). Here we wanted to check if this could be avoided in order to keep the field approach practical. The ddPCR approach was used as a control for the efficiency of the LAMP assay. Thus, the goal of this study was to design a LAMP assay that could specifically detect the invasive species *Pacifastacus leniusculus* and that would be sensitive enough to detect it in relatively small water samples (500 ml) that could be easily sampled and handled in eDNA-based field detection.

This could be of particular importance, as invasive crayfish are an ever-increasing concern for the conservation of freshwater habitats and especially streams^31^. The detection of their presence on a broad scale is crucial for planned mitigation, eradication or native crayfish species reintroduction. Thus, there is a need for the development of a specific tool for a reliable, cheap and quick monitoring that could enable, on a large scale, more efficient surveys than those operated through classical methods such as trapping. LAMP has the characteristics that could allow to achieve these goals.

## Results

### ddPCR *P. leniusculus* detection and DNA quantification

**Figure 1:**
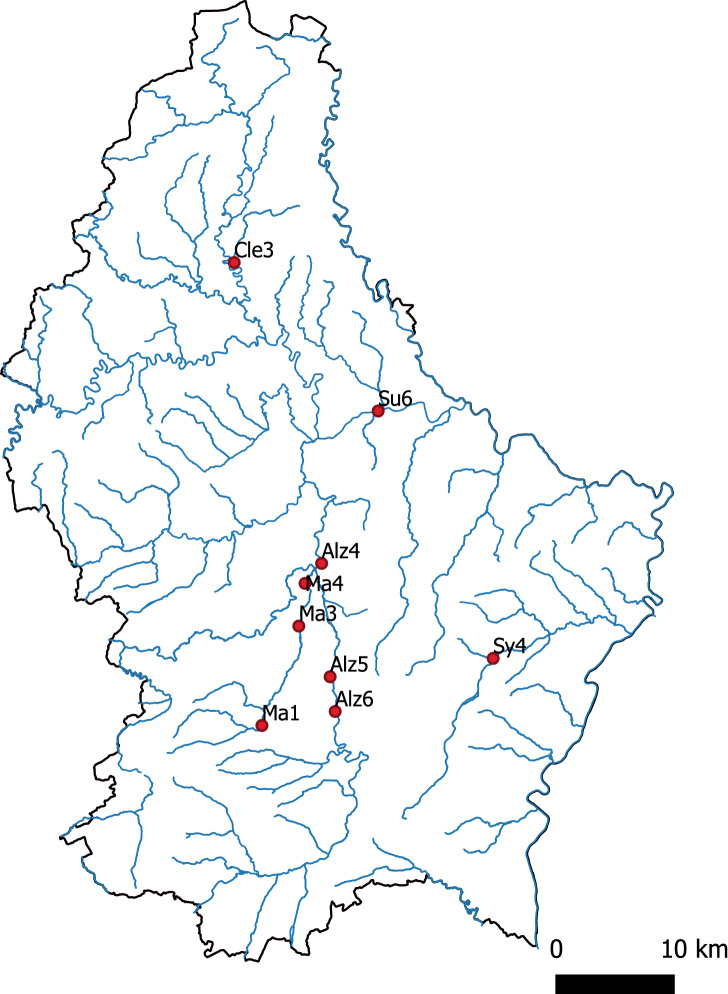
Map of the sampling sites in five rivers of Luxembourg (Cle3 = Clerve, Su6 = Sûre, Alz4-6 = Alzette, Ma1-3 = Mamer, Sy4 = Syre).

The different samples were analyzed, and the absolute concentration of the target DNA for each of the 90 replicates from the nine targeted sites was measured (Fig. 1, Table 1). The mean number of droplets generated was 18,021, and all amplification products were rePCRed and sequenced. The resulting sequences were found specific i.e. matching *P. leniusculus* haplotypes from Genbank and deposited on BOLD (ranging from 95.8 to 100% identity). No negative control, either field, extraction or amplification ones, yielded amplification. The concentrations measured ranged from 1.26 to 126 copies/µl (Supplementary Table S1). All sites were found positive for the presence of *P. leniusculus* with ddPCR.

**Table 1:** eDNA sampling sites and genomic DNA dilutions processed. Numbers in brackets are the number of repetitions (i.e. series of 5 technical replicates).

Site/dilution name	Number of LAMP dependent technical replicates	Number of LAMP independent technical replicates
Alz4	50 (10)	5(1)
Alz5	50 (10)	5(1)
Alz6	50 (10)	
Ma1	50 (10)	15(3)
Ma3	55 (10)	10(1)
Ma4	50 (10)	
Cle3	50 (10)	5(1)
Su6	50 (10)	10(2)
Sy4	50 (10)	5(1)
d1 (15 ng/µl)	10 (2)	10 (2)
d2 (1.5 ng/µl)	10 (2)	10 (2)
d3 (150 pg/µl)	10 (2)	10 (2)
d4 (15 pg/µl)	10 (2)	10 (2)
d5 (1.5 pg/µl)	10 (2)	10 (2)
d6 (150 fg/µl)	30 (6)	30 (6)
d7 (15 fg/µl)	10 (2)	10 (2)
d8 (1.5 fg/µl)	10 (2)	10 (2)

### Specificity and sensitivity tests

No amplifications were obtained from *F. limosus* and *A. astacus* DNA extracts with the LAMP assay designed for this study (Table 2).

**Table 2:** *P. leniusculus* LAMP primers.

Primer	Sequence (5'–3')
Pacif-F3	ACTAGAGGAATAGTTGAAAGAGG
Pacif-B3	GGACTGCTGTAATAAATACAGAT
Pacif-FIP	TCCTAAATCAACAGAAGCCCCTGCATTTTTGGATGAACTGTTTATCCTCCT
Pacif-BIP	TTCACTTCATTTAGCGGGTGTTTCTTTTTGATCTATAGTTATWCCTRCCCTTCG
Pacif-LF	TGAGCAATAGCCGCTGCT
Pacif-LB	GGGGCTGTAAATTTTATAACTACAGCTA

**Figure 2:**
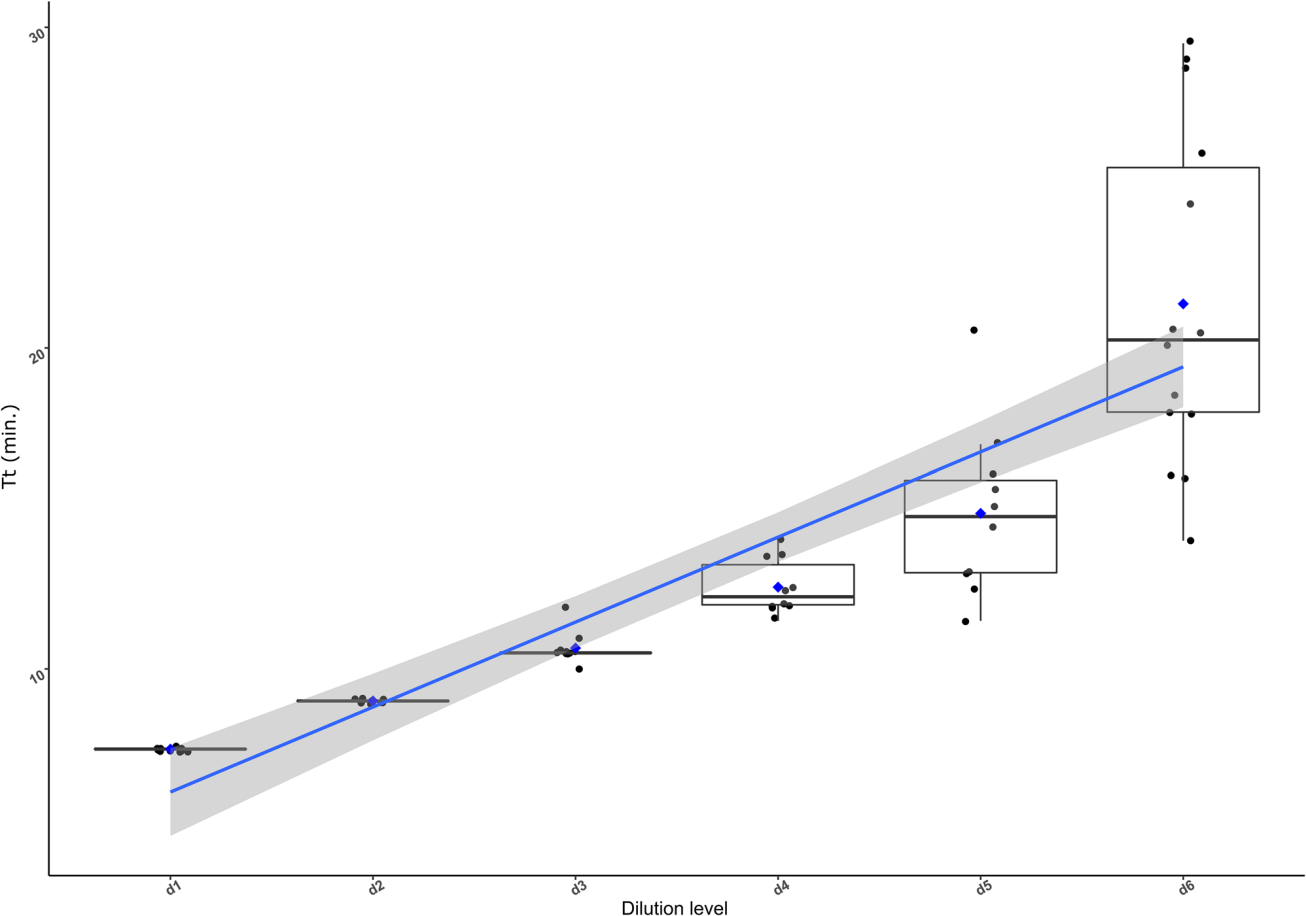
Correlation (blue line) and confidence interval (95%, gray shade) between the Time to threshold (Tt) obtained in LAMP assays in minutes and the decimal dilution levels. Boxplots represent the dispersion of the Tt measurements.

For the decimal dilutions of genomic DNA, a clear correlation was found between the Time to threshold (Tt) and the DNA concentration (R = 0.84, p < 2.26e−16, Fig. 2). No successful amplification was obtained beyond the dilution level d6 (150 pg/µl).

### Test of the dependent/independent technical replicates approaches

**Figure 3:**
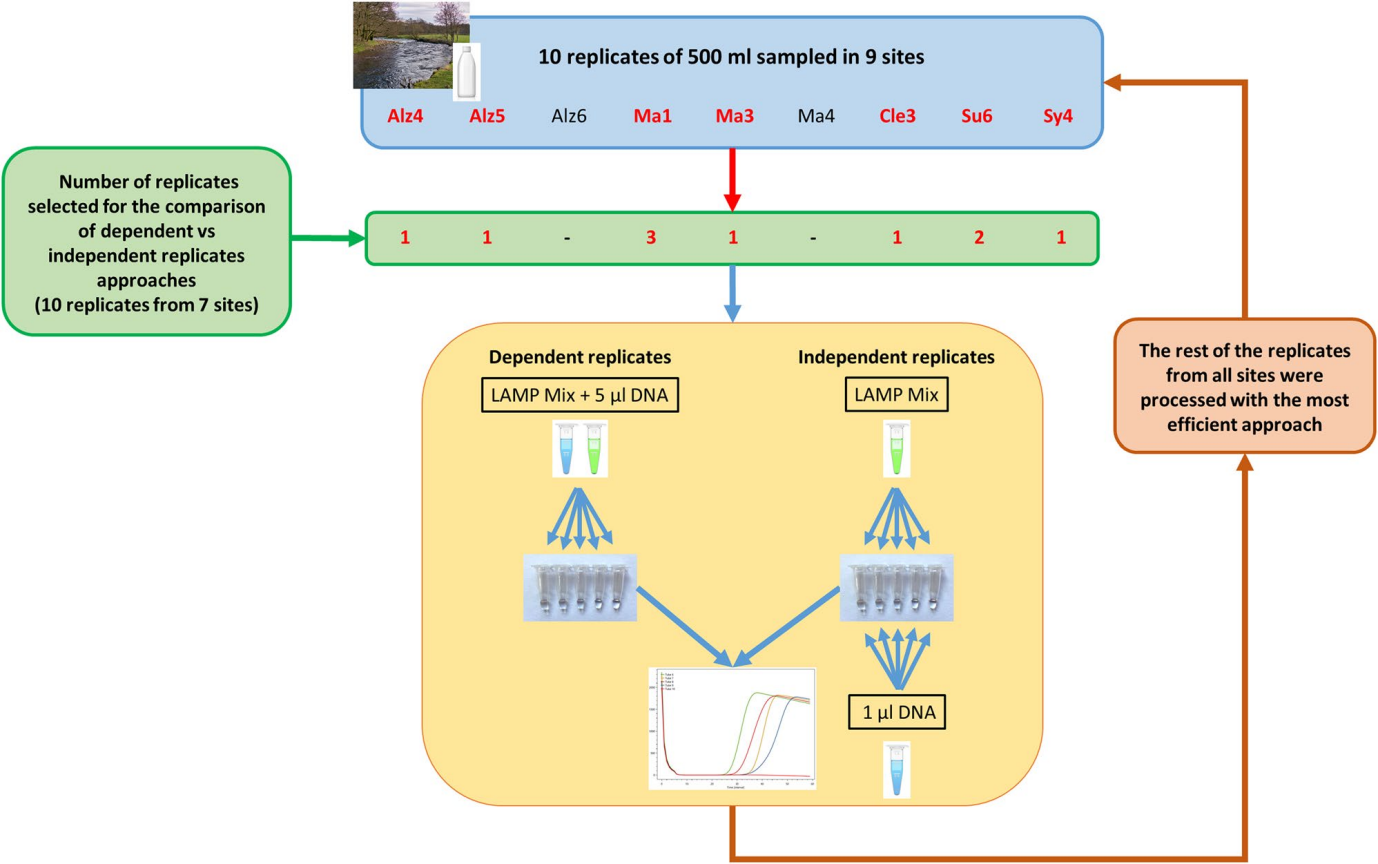
Schematic representation of the workflow for eDNA sampling and comparison between the independent and dependent replicates LAMP approaches.

In nine out of ten site replicates tested (from seven sites, Fig. 3), positive detection events were obtained either with the dependent or the independent replicates approach (Table 3). However, in five sites, the number of successes obtained was higher with dependent replicates (Table 3). One of the site replicates tested (Ma3-5) particularly illustrated the benefits of the dependent technical replicates approach with only two positive results out of ten (two series of five replicates) for the independent replicates, while the dependent replicate approach yielded nine positive detection events out of ten assays (Table 3).

**Table 3:** Number of positive and negative LAMP detection events in eDNA samples and in genomic DNA extract dilution d6 (150 pg/µl) for both the independent and independent technical replicates approaches.

	Alz4-1	Alz5-10	Cle3-7	Ma1-1	Ma1-2	Ma1-3	Ma3-5	Su6-2	Su6-8	Sy4-7	Total eDNA	d6	Total
Independent positive	2	1	1	4	4	3	2	1	2	3	23	5	28
Independent negative	3	4	4	1	1	2	8	4	3	2	32	25	57
Dependent positive	1	1	5	4	5	3	9	3	2	4	37	13	50
Dependent negative	4	4	0	1	0	2	1	2	3	1	18	17	35

**Figure 4:**
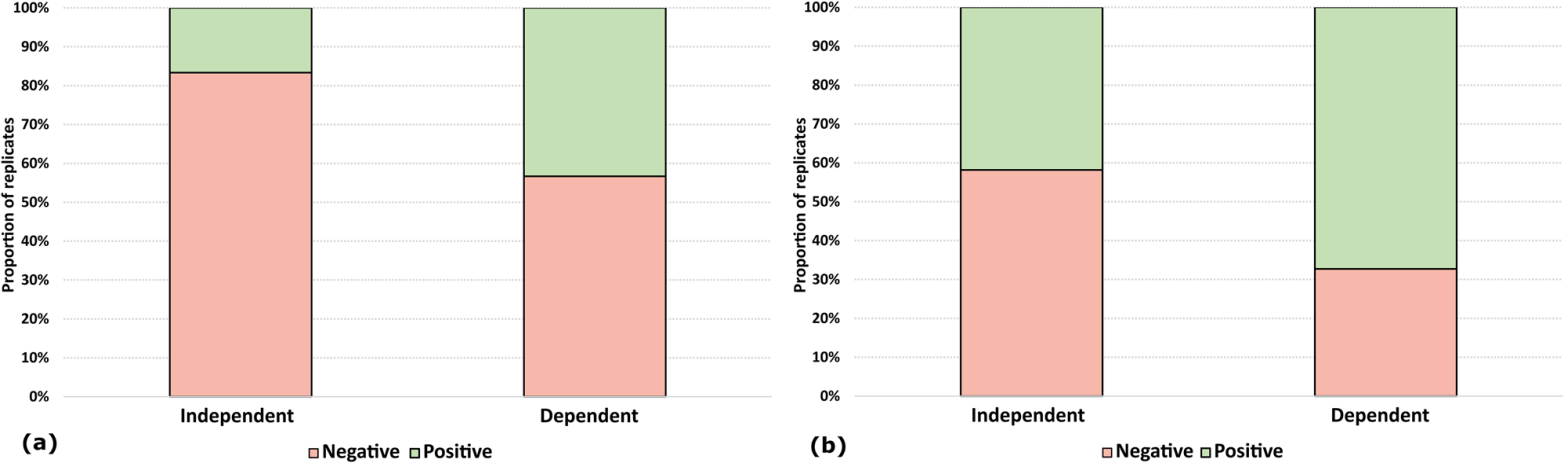
Cumulative histograms representing positive and negative events in the LAMP assay for **(a)** d6 genomic DNA dilution and **(b)** eDNA samples.

Globally, the dependent replicates approach was found more efficient either for d6 genomic DNA dilution (26.67% more positive replicates, X-squared = 5.0794, df = 1, p = 0.02421, Fig. 4a, Table 3) or eDNA samples (25.45% more positive replicates, X-squared = 7.1867, df = 1, p = 0.007345, Fig. 4b, Table 3). Moreover, in eDNA samples, the dependent technical replicates approach allowed a significant decrease in Tt (Pairwise Wilcoxon test, Holm correction, p = 0.004).

### Inhibition

**Figure 5:**
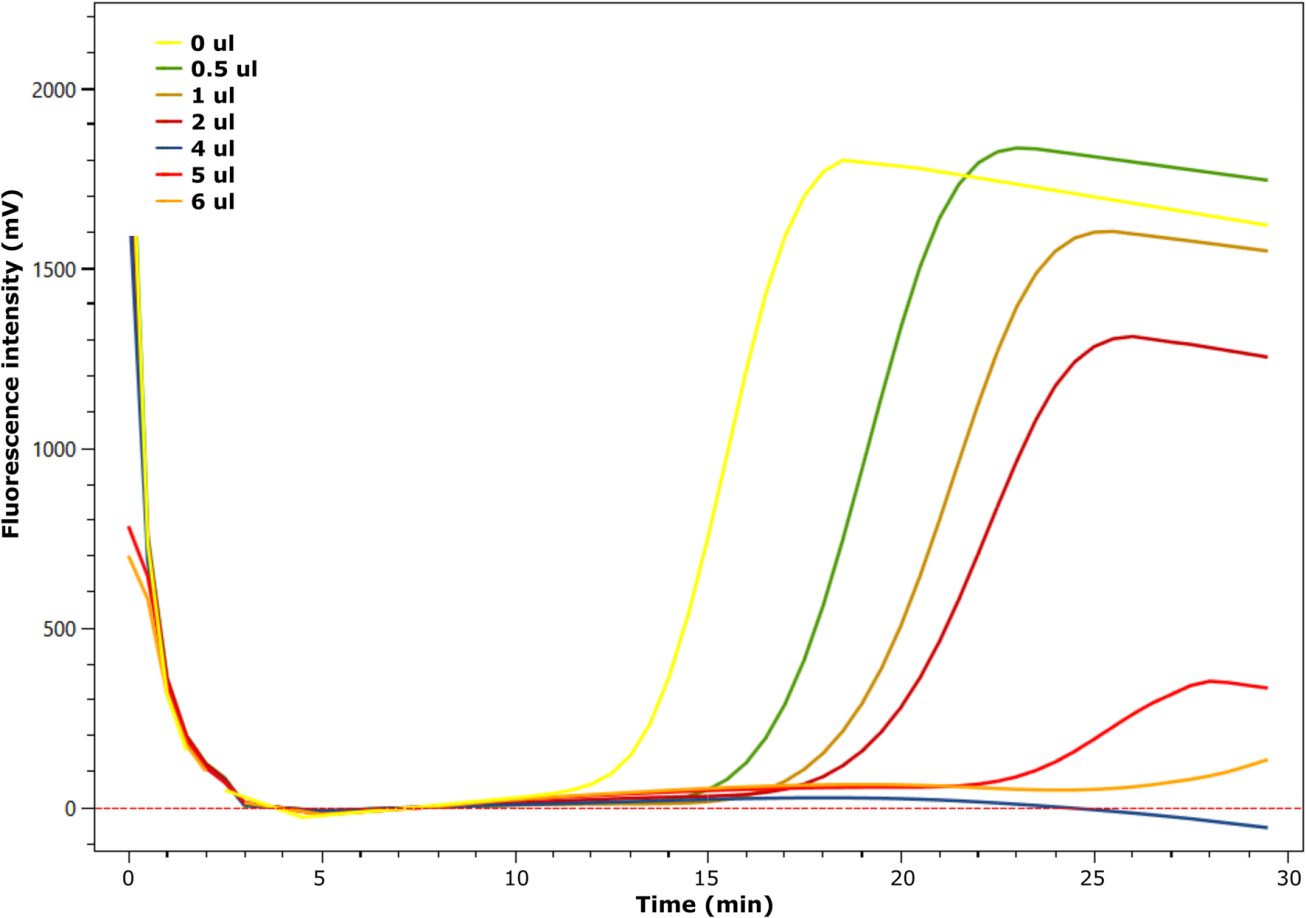
Inhibition effect on the LAMP assay targeted at the d3 dilution level (0.15 ng/µl) with the addition of an increasing amount of eDNA sample extract (Alzette 5–2) from 0 to 6 µl.

A clear inhibition effect on the LAMP assay was found with an increase in the Tt and a decrease in the fluorescence level when the volume of DNA extract from the Alzette river increased (Fig. 5).

### eDNA-based LAMP detection and comparison with ddPCR results

As it was found to perform better, the dependent technical replicates approach was applied to the rest of the eDNA samples. The LAMP assay designed for this study allowed the detection of *P. leniusculus* in all the sampled sites but in site Ma4. The correlation between Tt and DNA concentrations, found in serial dilutions of genomic DNA, was not retrieved with the eDNA samples (R = 0.15, p = 0.096).

ddPCR was chosen as a quantitative reference for template concentration i.e. COI barcode fragment from which both ddPCR and LAMP primers were designed from. Only a weak correlation was found between the DNA concentrations measured with ddPCR and the number of successful LAMP replicates (R = 0.16, p = 0.032—the seven DNA concentrations over 50 copies/µl were excluded from this analysis in order to avoid the constraint from the limited number of LAMP technical replicates (5) that would have artificially increased correlation statistics).

Detection-wise, the comparison of ddPCR with LAMP results showed that the LAMP assay designed here enables the target species detection in eight sites out of the nine sampled (no detection in site Ma4). Detection was possible from target concentration as low as 1.26 copies/µl. However, samples from three sites (Ma4, Alz5 and Alz6), exhibiting higher concentrations (ranging from 1.35 to 17.78 copies/µl), yielded no amplification. This was probably due to higher inhibitors contents.

No significant difference either in eDNA concentration measured by ddPCR or in success number for the LAMP assay was found between the replicates sampled at 30 cm and 1.50 m from the river bank (Pairwise Wilcoxon test, Holm correction, respectively p = 0.88 and p = 0.76).

## Discussion

### An efficient technique for eDNA in lotic environments

This study allowed to design a specific LAMP assay for the invasive crayfish species *P. leniusculus*. This assay proved sensitive enough to be used on eDNA extracts from 500 ml water samples, which, from ddPCR measurements, exhibited only a few copies. Moreover, it allowed to test a new approach employing dependent technical replicates which resulted in a higher level of detection efficiency for the present assay. Such a technique could be applied to other LAMP assays and might improve their sensitivity.

A previous study on lotic environments targeting *Dressenia* species^23^ proved LAMP to be efficient in detecting invasive organisms in this type of habitat. However, mussel species release very high numbers of eggs in the water (from 40 000 to one million per female) and, accordingly, also exhibit high larval abundances^26^. This likely produces more abundant DNA release than crayfish and, more generally, crustaceans, which are known to shed much lower amounts of DNA and in which larvae are not dispersed into the environment^27,28,32^. Here, we showed that LAMP assays could be used in lotic environments to detect the presence of such low DNA shedding organisms.

### Two signal attenuation factors: inhibition and dilution

Two main factors interfered with the eDNA-based detection in this assay. First, inhibition, originating from various inhibitors (e.g. humic and fulvic acids) carried over through the DNA extraction step can inhibit amplification. Even though Bst polymerases were proven to be less prone to inhibition^33,34^, inhibition of LAMP assays was previously reported in several studies (e.g. ^35–37^) and was proven to be inhibitor concentration-dependent^38^. Here, it impacted the efficiency of the reaction by (1) increasing the Tt, even from a low volume of extract containing inhibitors (0.5 µl), and (2) decreasing the fluorescence level until complete obliteration of the signal with a large amount of extract (5–6 µl). Combined with low concentration target DNA, this effect can be particularly important and causes false negative results, as shown at site Ma4: although the concentrations measured by ddPCR were comparable to those at site Alz5, the LAMP assay yielded no positive results. At the Ma4 site, the presence of a lot of suspended sediments in the water column prevented the complete filtering of the 500 ml replicates, probably both reducing the amount of DNA captured and bringing in high amounts inhibitors. This was also shown in a previous eDNA aquatic study using a LAMP assay in lotic environments, where direct amplification of concentrated samples yielded much fewer positive detection events than samples processed through classical DNA extraction where at least part of the inhibitors was filtered out^23^.

The second factor impacting the signal is dilution. It is crucial for eDNA-based detection studies as it can drastically reduce an already weak signal produced by low-density populations^39^. This concentration depletion challenges the consistency of DNA amplifications through the stochasticity effect it brings in. Here, we showed that countermeasures could be applied to mitigate this effect through the use of dependent technical replicates that significantly improved the LAMP assay sensitivity. This could be due to the fact that higher sampled volumes of DNA extracts with a subsequent homogenate distribution in reaction mix guaranties better chances to capture enough copies to allow amplification. By comparison, repeated smaller independent subsamples for the same total volume of DNA extract have lower probabilities of achieving this.

Even though, there is a degradation of the detection signal from this LAMP assay resulting from the combination of these two key factors. Either the weakness or the absence of correlation found between the DNA concentrations measured with ddPCR and, respectively, the number of LAMP positives and Tt values showed the uneven prominence of these two factors among sites. This can lead to false negatives (cf. Ma4) but also preclude further use of these metrics as a proxy for DNA concentration and thus any possible estimate of population abundance or biomass.

### Interest for invasive species monitoring

This *P. leniusculus*-specific assay, allied with the simplicity of the LAMP technique, i.e., a premade mix, a single temperature amplification, a possible colorimetric detection along with direct amplification approaches, has the potential to be used on a broad scale in the field or in rudimentary labs by minimally trained agents from governmental agencies but also in citizen science operations^23^. In both cases, ready-to-use kits could be operated by agents or volunteers that could further report detections through internet portals or smartphone applications^23^. This could enable a significant drop in the cost and efforts needed for the systematic monitoring of *P. leniusculus* in aquatic ecosystems.

## Conclusion

In contrast to previous targets of eDNA LAMP assays in lotic environments, crustaceans such as crayfish have less dense populations and shed lower amounts of DNA per individual^27,28^. This, combined with the high dilution factor and the inhibitor content that can be found in streams, could have possibly prevented the detection of these organisms with DNA-based LAMP methods. Here, we showed, through a species-specific experiment targeted at *P. leniusculus*, that LAMP assays can overcome these difficulties to produce reliable and sensitive detection.

Every year, massive amount of money and efforts are deployed in order to monitor invasive crayfish species in several countries. LAMP assays applied to eDNA samples could be a cheap, sensitive and quick alternative to classical monitoring actions based on trapping. Even if less sensitive and more prone to inhibition, eDNA-based LAMP assays could also be a less costly alternative to more sophisticated eDNA-based detection techniques, such as ddPCR, for broad-scale presence/absence surveillance networks. Applied to incoming invasive crayfish species in different geographic areas, it could contribute to enhance early warning systems efficiency.

## Materials and methods

### Crayfish material

*Pacifastacus leniusculus* and *Faxionus limosus* specimens were fished in the Alzette river (Hesperange Park lat: 49.57070, long: 6.15697) and in the Moselle river near Matchum (lat: 49.63555, long: 6.42951) in Luxembourg. *Astacus astacus* specimens were obtained from the Lingten fish breeding facility of the Luxembourgish Water Agency (AGE). Genomic DNA was extracted from subsampled leg’s muscle tissue from these specimens with a Qiagen DNeasy Blood & Tissue Kit following the manufacturer’s instructions.

### Water sampling

Nine sites in five rivers (Alzette, Clerve, Sure, Syre and Mamer—Table 1, Fig. 1) were sampled with 10 replicates of 500 ml water each. Each replicate was sampled with a 500 ml screw cap PET bottle. Five replicates were taken at 30 cm from the bank and five at 1.50 m. On each line (30 cm and 1.50 m), replicates were distant of 50 cm of each other. Sampling took place during the mating season, i.e., the first half of October, which is also a season when heavy rains have not yet overcharged the targeted rivers. These conditions were chosen in order to maximize the eDNA concentration level and thus detection probability.

Each water sample (kept for a maximum of 3 h at 4 °C before processing) was filtered with a peristaltic pump (Masterflex L/S Standard Pump Head) connected to a column driller (500 W) through a 0.45 µm nitrocellulose membrane (Nalgene analytical funnel). The resulting filter was immediately stored in 800 µl ATL Qiagen lysis buffer at 4 °C in 1.5 ml microfuge tubes and then frozen at −20 °C back in the lab. For each site, a negative control was established to detect any possible cross-contamination: a 500 ml bottle of distilled water was brought into the field and underwent the same storage and filtration process as the samples.

### DNA extraction

Filters were extracted for DNA using a Qiagen DNeasy Blood & Tissue Kit with a volume-adapted protocol: after a thorough shredding of the filter directly in the tube with clean scissors, 80 µl ProK was added to the lysis buffer used for filter preservation. After an overnight 56 °C incubation, 600 µl of the lysis solution was recovered and mixed with 600 µl AL Qiagen lysis buffer (10 min incubation at 56 °C) and 600 µl ethanol. Subsequently, after homogenization by mixing , the solution was transferred to Qiagen DNeasy 96 plates. The rest of the protocol followed the manufacturer’s instructions. DNA extracts were eluted in 100 µl of 56 °C warmed AE Qiagen elution buffer. In order to monitor any potential sample cross contamination, extraction negative controls (ATL buffer with proteinase K) were extracted along with each series of samples.

### Lamp assay

#### Primer design

A consensus sequence from 43 COI barcoding fragment sequences of *P. leniusculus* from GenBank along with 126 COI barcoding fragment sequences produced from the Luxembourgish populations and accessible in the public dataset DS-CRAYLUX (DOI:10.5883/DS-CRAYLUX) on BOLD (https://www.boldsystems.org/) (GenBank accessions: ON058995 - ON059120) was used to design species-specific primers. Six pairs of primers FIP/BIP (inner primers), F3/B3 (outer primer), LF, and LB (Loop primers) (Table 2) were designed with the online software PrimerExplorer V5 (https://primerexplorer.jp).

### Amplification conditions

Each reaction mix was composed of 12.5 µl LavaLAMP mastermix (Lucigen) with 2.5 µM Syto16 fluorescent stain, 16 µM FIP/BIP, 2 µM F3/B3 and 8 µM LP/LF, complemented with water to 23 µl. One microliter template or water was added for detection and negative control assays, respectively. The reactions were incubated at 68 °C for 30 min and terminated at 95 °C for 5 min with an ESEQuant TS2 device (Qiagen), which measured the fluorescence of the assay every 30 s intervals. The Time to threshold (Tt) was obtained with a threshold value set to 200 units of fluorescence and interpreted with the software ESQuant TS2 Studio (Qiagen). A negative control was included in each run.

### Specificity and sensitivity tests

The assay was tested for specificity with genomic DNA extracts from the other species of crayfish that could be encountered in Luxembourgish rivers: *Faxionus limosus* and *Astacus astacus*. Ten replicates were run for each of the two species.

A serial dilution of genomic DNA (from d1 to d8, respectively from 15 ng/µl to 1.5 fg/µl) was used to evaluate the sensitivity of the assay. Ten technical replicates were amplified for each dilution, and 20 additional ones were processed for the last dilution level exhibiting a positive result i.e. 30 replicates total for the last dilution with positive detection events (Table 1).

In order to obtain further improvement mitigating the inerrant stochasticity of the amplification reaction due to low concentration templates, two methods were compared for the lowest concentration that enabled detection: (1) independent technical replicates with 1 µl DNA in each reaction volume i.e. the currently used method in LAMP assays and (2) dependent technical replicates redistributed from 5 µl DNA mixed in five reaction mix volumes (Fig. 3). For each method, 30 technical replicates were processed.

### Inhibition

The inhibition effect was tested through a series of assays in which a *P. leniusculus* DNA extract (d3 = 0.15 ng/µl) was successively spiked with an increasing volume of a river eDNA extract (0, 0.5, 1, 2, 4, 5, 6 µl). The eDNA extract was chosen from one of the three Alz5 site’s replicates (out of ten) as this site was one of the three ones suspected of higher inhibition content with Ma4 and Alz6 (Table S1). The increasing amount of eDNA extract replaced an equivalent volume of water in the reaction mix.

### eDNA extracts LAMP detection

The efficiency of the five independent and dependent technical replicates approaches was tested on the ten site replicates from seven sites (Table 1, Fig. 3). In total, 55 technical replicates were processed for each of the two methods i.e. the dependent and the independent replicates approaches (Table 1, Fig. 3). The most efficient approach was used to process the rest of the eDNA samples with five technical replicates for each site replicate.

### ddPCR

The extracts were processed for ddPCR and read on a Bio–Rad QX200 suite according to the manufacturer’s instructions (https://www.bio-rad.com/webroot/web/pdf/lsr/literature/Bulletin_6407.pdf). The evagreen mix was used along with the specific primer set previously designed for *P. leniusculus* ^40^. The PCR cycling program followed the manufacturer’s instructions with an annealing temperature of 60 °C and 50 cycles. The reaction mix was composed of 11 µl Evagreen Bio–Rad Supermix and 200 nM primer, completed with water to 21 µl and added to 1 µl template DNA or water as a negative control. Products from duplicates were recovered from emulsion according to Bio-Rad’s instructions (https://www.bio-rad.com/webroot/web/pdf/lsr/literature/Bulletin_6407.pdf), rePCRed using the same primer set at 0.25 µM concentration with 2 µl of 1/10 diluted recovered PCR products added to 15 µl of GoTaq mastermix (Promega) and completed with water to 25 µl. Temperature program was 95 °C for 3 min followed by 35 cycles of denaturation at 95 °C for 30 s, annealing at 60 °C for 30 s, extension at 72 °C for 60 s and a final extension at 72 °C for 5 min. The amplicons were purified with Agencourt AMPure XP and sequenced both directions with Big Dye terminator. A second purification with Agencourt CleanSEQ eliminated the excess dye-terminators. The sequencing was run on a Sanger sequencer ABI 3730 XL. The sequences obtained were aligned to the COI haplotypes present on Genbank and BOLD for specificity control.

The absolute concentration of the samples was recovered and compared with the Time to threshold (Tt) of LAMP assays for the corresponding sample.

### Statistics

Statistical analyses were conducted in the RStudio V1.2.5042 environment^41^, and graphs were plotted with the packages ggpubr and ggplot2^42^.

## Supplementary Information

Supplementary Table S1.

## Data Availability

The datasets generated and analyzed during the current study are available from the corresponding author on request.
