# Supplementary material for: eDNA-based detection of the invasive crayfish Pacifastacus leniusculus in streams with a LAMP assay using dependent replicates to gain higher sensitivity
Source: Sci Rep. 2022 Apr 21;12:6553. doi: 10.1038/s41598-022-10545-w (PMC9023534; doi:10.1038/s41598-022-10545-w)
Supplement: Supplementary file 1 — Supplementary Table S1. [file 41598_2022_10545_MOESM1_ESM.docx]

Table S1 ddPCR absolute DNA concentration measurements, number of LAMP positive detection events and water volume filtered for each replicates from each sites.
